# Development of a prediction model for infant hospitalisation and death using clinical features assessed by community health workers during routine postnatal home visits in Dhaka, Bangladesh

**DOI:** 10.1136/bmjpo-2026-004626

**Published:** 2026-06-25

**Authors:** Alastair Fung, Marimuthu Sappani, Cole Heasley, Chun-Yuan Chen, Shaun K Morris, Peter J Gill, Diego G Bassani, Davidson H Hamer, Prakesh S Shah, S M Abdul Gaffar, Sultana Yeasmin, Shafiqul A Sarker, Shamima Sultana, Joseph Beyene, Daniel E Roth

**Affiliations:** 1 Department of Paediatrics, The Hospital for Sick Children, University of Toronto, Toronto, Ontario, Canada; 2 Centre for Global Child Health, The Hospital for Sick Children, Toronto, Ontario, Canada; 3 Dalla Lana School of Public Health, University of Toronto, Toronto, Ontario, Canada; 4 Child Health Evaluative Sciences, The Hospital for Sick Children Research Institute, Toronto, Ontario, Canada; 5 Department of Health Research Methods, Evidence and Impact, Faculty of Health Sciences, McMaster University, Hamilton, Ontario, Canada; 6 Department of Global Health, Boston University School of Public Health, Boston, Massachusetts, USA; 7 Section of Infectious Diseases, Boston University Chobanian and Avedisian School of Medicine, Boston, Massachusetts, USA; 8 Department of Pediatrics, Mount Sinai Hospital, Toronto, Ontario, Canada; 9 International Centre for Diarrhoeal Disease Research, Dhaka, Bangladesh

**Keywords:** Infant, Epidemiology, Low and Middle Income Countries, Machine Learning, Child Health

## Abstract

**Background:**

To improve upon the WHO 8 danger signs used to identify young infants (<2 months) requiring referral during community health worker (CHW) home visits, aggregative features (eg, cumulative visits with fever) rather than visit-specific features (eg, fever at a single visit), and a machine learning random forest model, may enhance predictive performance. Applying these approaches, we aimed to develop a prediction model for infant hospitalisation and/or death using CHW-assessed clinical features during home visits in Dhaka, Bangladesh.

**Methods:**

We analysed data from generally healthy infants prospectively enrolled at birth and assessed at 11 scheduled CHW visits from 3 to 60 days of age. To predict first hospitalisation or death, we developed two models—time-varying Cox regression and random forest—using the same candidate predictors (45 clinical features of which eight were WHO danger signs and 12 additional covariates) with aggregative features incorporated. We evaluated discrimination (C-statistic) and calibration (calibration plots). Performance was compared with a time-varying Cox model using only WHO danger signs.

**Results:**

Among 1906 infants, 176 (9.2%) had an event (173 hospitalisations, three deaths). The best-performing Cox model (C-statistic=0.71; 95% CI 0.68 to 0.75) consisting of three baseline covariates (any perinatal/delivery complication, umbilical cord care and gestational age) and four visit-specific clinical features (nasal congestion, cough, jaundice and skin rash) and a Cox model with these four features plus WHO danger signs (C-statistic=0.70; 95% CI 0.67 to 0.74) demonstrated higher discrimination than WHO danger signs alone (C-statistic=0.56; 95% CI 0.54 to 0.60), with similar calibration. A random forest model (42 predictors) was well-calibrated with comparable discrimination (C-statistic=0.69; 95% CI 0.64 to 0.73).

**Conclusions:**

Aggregative features and random forest did not outperform a time-varying Cox model using baseline covariates and visit-specific features. Among lower-risk infants, adding four features to WHO danger signs may improve predictive performance by capturing a broader spectrum of illnesses requiring hospitalisation.

**WHAT IS ALREADY KNOWN ON THIS TOPIC**

- During community health worker (CHW) home visit assessments of young infants (<2 months), use of WHO-recommended danger signs to predict hospitalisation and/or death may have limited sensitivity and may miss cases of severe illness requiring referral.
- Summarising repeated assessments of clinical features during sequential home visits as aggregative predictors (eg, cumulative visits with fever) rather than visit-specific predictors (eg, fever at a single visit), and machine learning models such as random forest, have not been previously evaluated for prediction of infant hospitalisation and/or death and may improve predictive performance compared with WHO danger signs.

**WHAT THIS STUDY ADDS**

- Random forest, and use of aggregative predictors in both a random forest model and a time-varying Cox model, did not improve prediction of infant hospitalisation and/or death during CHW routine home visits compared with a time-varying Cox model consisting of baseline covariates and visit-specific clinical features.
- Adding four visit-specific clinical features to the WHO danger signs improved prediction of hospitalisation and/or death during CHW routine home visit assessments of young infants born generally healthy in an urban setting.

**HOW THIS STUDY MIGHT AFFECT RESEARCH, PRACTICE OR POLICY**

- The findings support future research evaluating whether adding visit-specific clinical features to the WHO danger signs algorithm can improve identification of infants needing referral across diverse settings and with varying baseline risks.

## Introduction

 In 2024, 2.3 million infants worldwide died in the first month of age, with the highest burden in sub-Saharan Africa and central/southern Asia.^1^ In low- and middle-income countries (LMICs), home-based postnatal care interventions, including community health worker (CHW) home visit assessments and referrals of sick newborns, have been shown to reduce neonatal mortality.^2 3^ Infections are a leading cause of neonatal mortality, with sepsis accounting for 20% of neonatal deaths.^4^ More than half of sepsis-related neonatal deaths in LMICs occur after the first week of age.^5^ Therefore, home-based identification of life-threatening illnesses after the first week of age and referral to hospital are critical to reducing infant morbidity and mortality in LMICs.

The WHO recommends assessment of eight clinical signs (‘WHO danger signs’) by first-level health workers, including CHWs, during postnatal home visits to identify sick young infants (<2 months): not feeding well, history of convulsions, fast breathing (≥60 breaths per minute), severe chest indrawing, no spontaneous movement, fever (≥37.5 °C), low body temperature (<35.5°C) and any jaundice in the first 24 hours of life or yellow palms and soles at any age.^6 7^ Identification of any one sign indicates need for referral for further evaluation.^7^

In a recent secondary analysis of a birth cohort in Bangladesh, India and Pakistan (ANISA), each of seven signs of possible serious bacterial infection (pSBI; seven of the eight WHO danger signs, excluding jaundice) assessed by CHWs in young infants during scheduled home visits was significantly associated with mortality.^8^ However, relying solely on WHO danger signs during routine home visits presents potential challenges. First, WHO danger signs were originally derived among infants brought to health facilities due to caregiver concern.^9^ These infants likely had higher pretest probability of requiring hospital-based care than infants assessed during routine home visits. Second, external validation of the WHO eight danger signs algorithm in a routine home visit setting^10^ was limited to a small sample size (n=395) during the first week of age. Third, the algorithm had high specificity (95%) but lower sensitivity (69%) for physician-assessed severe illness requiring referral, raising concern that its use may miss cases.^10^ Fourth, although WHO guidelines recommend multiple CHW home visits, the current algorithm considers the data from each visit in isolation and does not utilise accumulated information. The WHO danger signs are employed as *visit-specific* time-varying predictors (eg, occurrence of fever (yes/no) at each visit). However, beyond the infant’s most recent visit, it is also possible to summarise repeated assessments of clinical signs at prior visits as *aggregative* time-varying predictors (eg, cumulative number of visits with fever). Aggregative time-varying predictors may better reflect illness trajectory and improve predictive performance compared with a model based on the WHO danger signs, while acknowledging that aggregative time-varying predictors depend on repeated assessments and retention of data from prior visits which may affect feasibility. Finally, clinical sign-based algorithms, including the WHO danger signs algorithm, to predict sepsis and death in young infants have largely been developed using traditional regression.^11 12^ Machine learning models such as random forest have been shown to outperform traditional regression approaches to identify SBI in febrile young infants presenting to emergency departments in a high-income country.^13^ A recent scoping review of clinical prediction models to diagnose neonatal sepsis in LMICs identified some machine learning models.^14^ However, these models rely on laboratory test predictors and were developed for use in neonatal intensive care units. To the best of our knowledge, machine learning models to predict severe illnesses and death in infants using clinical features assessed by CHWs during routine home visits remain unexplored. Advantages of machine learning models such as random forest over traditional regression approaches are that they can flexibly model non-linear relationships between predictors and an outcome, naturally account for interactions among predictors and handle a larger number of predictors without being prone to overfitting.^15^

We hypothesised that the predictive performance of the WHO danger signs algorithm could be improved in two ways: first, by incorporating aggregative time-varying predictors that summarise repeated assessments across home visits; and second, by applying a machine learning approach such as random forest. Therefore, using two modelling methods—time-varying Cox regression and random forest—each applied to the same set of CHW-assessed candidate clinical features, including aggregative time-varying features, we aimed to develop a prediction model for hospitalisation and/or death among young infants during routine home visits in Dhaka, Bangladesh.

## Methods

### Study design and data source

This was a secondary analysis of data from the Synbiotics for the Early Prevention of Severe Infections in Infants (SEPSiS) observational cohort study (NCT04012190).^16^ From 25 November 2020 to 18 February 2022, mother-infant pairs were screened for eligibility at two government healthcare facilities in Dhaka: Maternal and Child Health Training Institute (Azimpur) and Mohammadpur Fertility Services and Training Centre. Infants born generally healthy were enrolled between day 0 (birth) and day 4 of age. This cohort (n=1939) has been described separately^17^ and detailed inclusion/exclusion criteria are in online supplemental panel S1. Community health research workers (CHRWs) conducted infant assessments at up to 11 scheduled in-person home visits at 3- and 6 days post-enrolment, and on days 10, 14, 21, 28, 35, 42, 49, 56 and 60 postnatal age. If in-person visits were not feasible, assessments were attempted by telephone. Infants with at least one in-person CHRW home visit after enrolment were included in this analysis. The observation period was from the first CHRW home visit to day 67 of age, allowing predictors ascertained up to the 60-day visit to predict events up to day 67.

This study followed the TRIPOD+AI (Transparent Reporting of a multivariable prediction model for Individual Prognosis Or Diagnosis+Artificial Intelligence) guidelines.^18^ Caregivers/public were not involved in study design, conduct or dissemination.

### Outcome

The outcome was time to an event, defined as either first hospitalisation and/or death, following the first CHRW visit up to day 67 of age. If an infant was hospitalised and subsequently died, this counted as one event at time of hospitalisation. If an infant had multiple hospitalisations, only the first hospitalisation was included. Hospitalisations recommended by physicians but declined by caregivers were included. Hospitalisations prior to the first CHRW visit were excluded but these infants were included in the analysis following that visit. Two infants died before their first CHRW in-person visit and were excluded. Hospitalisations for elective surgeries or trauma were excluded. Physicians recommending hospitalisation were not study staff, but they may have been informed by study physicians about CHRW findings.

Hospitalisations were identified through multiple pathways, including identification by study medical officers for admissions to study hospitals, notification mechanisms for admissions to non-study hospitals and caregiver report at scheduled visits. Study medical officers assigned diagnostic labels based on the treating (non-study) physician’s diagnosis using predefined categories, with free-text entry permitted when needed. Deaths were captured as serious adverse events, either prospectively if death occurred during an admission to a study hospital or retrospectively via caregiver report at later visits or notification mechanisms if death occurred elsewhere. Verbal autopsy was performed by study physicians for all infant deaths and medically certified causes of death were obtained when available.

### Primary predictors

Primary candidate predictors were 45 clinical features (25 symptoms from caregiver history, 20 physical exam signs) assessed by CHRWs during scheduled home visits. These features were prespecified based on clinical relevance. Using a standardised checklist, trained CHRWs assessed symptoms and signs occurring on the scheduled visit day, in the last 7 days or since the last study visit (whichever was most recent). Predictor ascertainment was restricted to in-person visits, as physical examination signs could not be reliably assessed during telephone visits. Each of the 45 clinical features was operationalised as *visit-specific* (ie, based on data obtained at a single visit) and *aggregative* (ie, based on accumulated data from prior visits) time-varying predictors (online supplemental tables S1–S3). Candidate predictors included the WHO eight danger signs (which, as used in the WHO danger signs algorithm, are visit-specific). Per the SEPSiS protocol, CHRW ascertainment of any WHO danger sign mandated referral to a study physician.

### Additional covariates

Additional covariates were included as predictors based on clinical judgement. Time-fixed additional covariates obtained at enrolment or the first scheduled visit included maternal age, maternal education, antenatal care, infant sex, gestational age at birth, preterm birth (<37 weeks), birth weight-for-gestational age Z-score, umbilical cord care and any perinatal/delivery complication. Time-varying additional covariates included maternal postpartum substance use, exclusive breastfeeding status and systemic antibiotic administration on the visit day, in the last 7 days or since the last visit (whichever was most recent). Details on the ascertainment and derivation of additional covariates can be found in online supplemental panel S2.

### Sample size and missing data

Sample size was fixed based on available data from the SEPSiS study eligible for this secondary analysis. Missing values of clinical features were planned to be imputed, if necessary, using multiple imputation.^19^ However, given minimal missingness of clinical features (1.2% to 1.4%), the risk of bias due to missing data was considered low, and models were developed using complete case analysis.

### Statistical analysis

Demographic characteristics were described using frequencies and percentages for categorical variables, and continuous variables were summarised by means and SD, or medians and IQRs.

#### Variable selection using unadjusted analyses

Variables were selected for inclusion in prediction models based on clinical and statistical factors. Unadjusted time-varying Cox regression analyses were performed to estimate HRs with 95% CIs and p-values. Prevalence of each feature was also considered during predictor selection. In the primary analysis for the time-varying Cox model, clinical features with prevalence ≥1% were used. In the primary analysis for the random forest model, which can handle a larger number of predictors than a regression model while avoiding overfitting, clinical features with prevalence ≥0.1% (instead of ≥1%) were used. For both the time-varying Cox model and the random forest model, among all aggregative and visit-specific operationalisations for each candidate clinical feature (online supplemental tables S1–S3), the operationalisation with the lowest p-value<0.2 in unadjusted analyses^20^ was selected for multivariable analysis. Multicollinearity was assessed using the variance inflation factor (VIF) and variables with the highest VIFs ≥5 were sequentially removed until all VIFs were <5.^21^

#### Time-varying Cox model

Using predictors selected based on unadjusted analyses and multicollinearity, backward selection with a threshold p-value of <0.2 was used in a time-varying multivariable Cox regression analysis to derive a prediction model.^20^ After backward selection, additional covariates and clinical features with p-value<0.2 were reincluded in the model if this improved the C-statistic, or removed if this did not change the C-statistic, to achieve the most accurate and parsimonious model. Internal validation was done using fivefold cross-validation.^20^

Additional analyses included: (1) evaluating discrimination and calibration of a time-varying Cox model based on the WHO eight danger signs and a time-varying Cox model based on a single predictor denoting at least one danger sign; (2) adding a single predictor denoting at least one WHO danger sign to the best-performing Cox model in the primary analysis; (3) using only additional covariates and clinical features from caregiver history (excluding physical exam signs); (4) removing hospitalisations with a primary admission diagnosis of jaundice; (5) using a prevalence threshold of ≥0.1% instead of ≥1% for predictor inclusion in multivariable analysis and (6) after backward selection, not reincluding or removing additional covariates with p-value<0.2 from models if this improved discrimination. The rationale for the analysis excluding jaundice hospitalisations is that we observed a high proportion of hospitalisations for jaundice and a strong association between the jaundice predictor and these hospitalisations. We sought to evaluate whether model performance was retained for other clinically significant illnesses.

Sensitivity analyses included: (1) using a threshold p-value of <0.05 instead of <0.2 for backward selection; (2) excluding infants who ever had systemic antibiotic administration reported during routine home visit assessments; (3) including events up to 4 weeks after the last CHRW visit; (4) excluding hospitalisation events <48 hours duration; (5) imputing missingness of clinical features with last observation carried forward and (6) reducing the maximum number of scheduled visits.

Subgroup analyses included assessing discrimination of the best-performing model by (1) age group (0–28 days vs 28–60 days), (2) sex and (3) maternal education level.

#### Random forest model

Using the set of predictors selected based on unadjusted analyses and multicollinearity, we derived a random forest model that emulated the random forest for survival, longitudinal and multivariate data analysis (RF-SLAM) model developed by Wongvibulsin *et al* that can handle repeated measurements with time-varying predictors.^22^ Details of RF-SLAM model development are provided in online supplemental panel S3.

In an additional analysis, predictors were selected using a prevalence threshold of ≥1%, instead of ≥0.1%, for selection for inclusion in the multivariable RF-SLAM analysis. A further analysis applied the RF-SLAM approach to the predictors included in the best-performing time-varying Cox model.

The performance of all models was evaluated using discrimination (C-statistic and time-dependent area under the receiver operating characteristic curve (AUC) with 95% CIs generated by bootstrapping) and calibration (visual assessment of calibration plots).^23^

All analyses were done using R V.4.4.0 software.^24^

## Results

**Figure 1:**
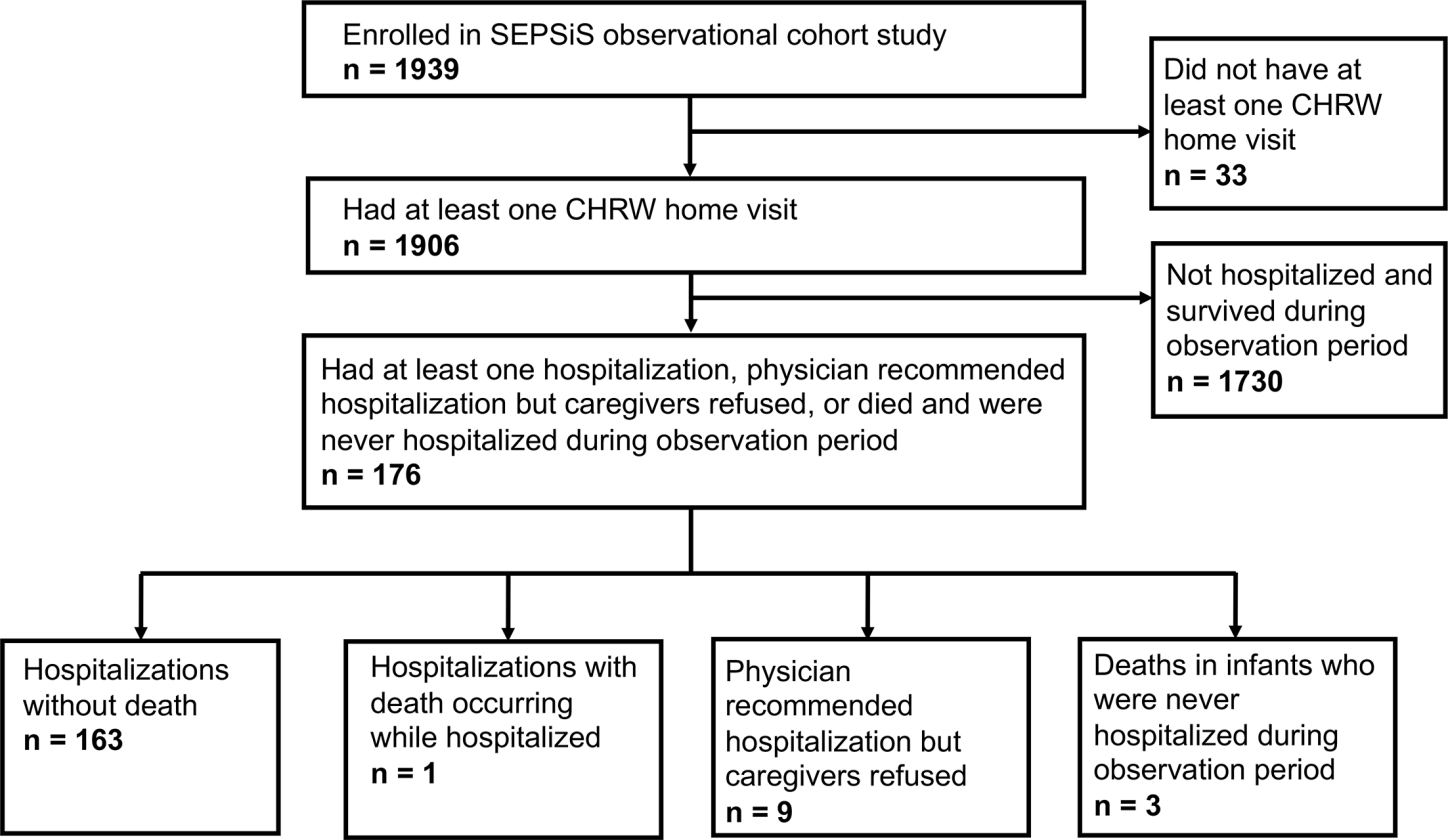
Study flow diagram. CHRW, community health research workers.

A total of 1906 infants had at least one CHRW home visit and were included (figure 1). Of these, 176 had a hospitalisation and/or death event (figure 1). Among nine infants for whom hospitalisation was recommended but declined by caregivers, all survived and two were subsequently hospitalised the following day. Diagnoses and causes of death are shown in online supplemental tables S4 and S5. CHRWs conducted 20 472 visits (82% in-person, 18% by telephone). The median (25th, 75th) number of visits (in-person and telephone) per infant was 11 (10, 11). Sixty-two per cent of events occurred during the first 4 weeks of age and the median (25th, 75th) interval between an event and the most recent CHRW visit was 2 (1, 5) days (online supplemental figures S1 and S2). Of the additional covariates (table 1), gestational age, any perinatal/delivery complication, umbilical cord care and exclusive breastfeeding status and systemic antibiotic administration since the last visit met the p-value<0.2 threshold and were included in multivariable analyses.

**Table 1:** Baseline characteristics and additional covariates and their associations with hospitalisation and/or death in unadjusted analyses

Characteristic	Overall (n=1906)	No hospitalisation or death (n=1730)	Hospitalised at least once or died without hospitalisation (n=176)	Unadjusted HR (95% CI)	P value
Maternal age (years), median (25th, 75th)*	24 (20, 27)	24 (20, 27)	23 (20, 27)	1.0 (0.97 to 1.0)	0.99
Maternal education, n (%)*					
None up to complete primary school	533 (28)	475 (27)	58 (33)	Reference	
Secondary incomplete	612 (32)	561 (33)	51 (29)	0.75 (0.52 to 1.1)	0.14
Secondary complete or higher	748 (40)	683 (40)	65 (37)	0.84 (0.59 to 1.2)	0.35
Received antenatal care, n (%)*	1845 (97)	1677 (97)	168 (96)	1.3 (0.41 to 4.0)	0.66
Female, n (%)*	996 (52)	910 (53)	86 (49)	0.87 (0.64 to 1.2)	0.34
Gestational age at birth (weeks), median (25th, 75th)†‡	39.1 (38.3, 40.1)	39.1 (38.3, 40.1)	38.9 (38.1, 39.9)	1.1 (1.0 to 1.2)	0.021
Preterm birth (<37 weeks GA), n (%)*	146 (7.7)	130 (7.5)	16 (9.1)	1.3 (0.76 to 2.1)	0.36
Birth weight-for-gestational age Z-score, median (25th, 75th)‡	−0.81 (−1.5, −0.11)	−0.81 (−1.5, −0.11)	−0.86 (−1.5, −0.096)	1.0 (0.91 to 1.2)	0.66
Any perinatal or delivery complication, n (%)*†§	107 (5.6)	93 (5.4)	14 (8.0)	1.6 (0.91 to 2.7)	0.11
Umbilical cord care†					
None	276 (15)	262 (15)	14 (8.0)	Reference	
Antiseptic	1139 (60)	1023 (59)	116 (66)	2.1 (1.2 to 3.6)	0.011
Antiseptic and other substance(s)¶	374 (20)	340 (20)	34 (19)	1.7 (0.91 to 3.2)	0.095
Other substance(s)¶	117 (6.1)	105 (6.1)	12 (6.8)	2.0 (0.90 to 4.2)	0.089
Exclusive breastfeeding status since the last study visit, n (%)*†**	1596 (84)	1455 (84)	141 (80)	0.72 (0.48 to 1.1)	0.10
Any maternal postpartum substance use†† since last study visit, n (%)* **	132 (7.0)	124 (7.2)	8 (4.6)	0.59 (0.26 to 1.4)	0.21
Systemic antibiotic administration since last study visit, n (%)†**	14 (0.73)	13 (0.75)	1 (0.57)	3.1 (2.0 to 5.0)	<0.001

Missingness for the above variables ranged from 0.68% to 2.7%.

* Total number of mothers n=1893.

† Met the p-value threshold of <0.2 and were selected as predictors in multivariable analyses.

‡ Unadjusted HR inverted so that lower value corresponds to higher risk.

§ Perinatal or delivery complication refers to any one or more of the following: maternal haemorrhage, premature rupture of membranes, prolonged rupture of membranes, foul smelling amniotic fluid, maternal fever, chorioamnionitis, maternal sepsis, fetal distress, sustained fetal bradycardia, sustained fetal tachycardia or other complication.

¶ Other substance refers to any one or more of the following: lotion, *desi ghee*/butter, *haldi* (turmeric powder), mustard seed oil, coconut oil, powder/wheat flour, antimony (*surmah*), *matti* (soil/clay), unknown.

** Time-varying variable. Proportions are based on data from first scheduled visit. Unadjusted HR and p-value account for data from all visits using time-varying Cox regression.

†† Postpartum substance use refers to use of any one or more of the following: smoked tobacco (cigarettes), Zarda (sweetened tobacco), tamak-pata (tobacco leaf), gul (dried and powdered tobacco), Pan (betel leaf), Supari (betel nut), Chuna (lime paste), unknown.

GA, gestational age.

Among the clinical features assessed by CHRWs, predictors selected using the p-value from unadjusted analyses and multicollinearity assessment for the primary analysis (lowest p-value<0.2 in unadjusted analysis, VIF<5, prevalence ≥1%) were all visit-specific time-varying operationalisations except for the cumulative sum of visits with red, oozing and/or swollen eyes up to the last visit, which was an aggregative time-varying predictor (online supplemental table S6). All other aggregative time-varying operationalisations had higher p-values, and HRs attenuated to the null, compared with corresponding visit-specific variables, and were therefore not carried forward to multivariable analysis (online supplemental table S7).

**Figure 2:**
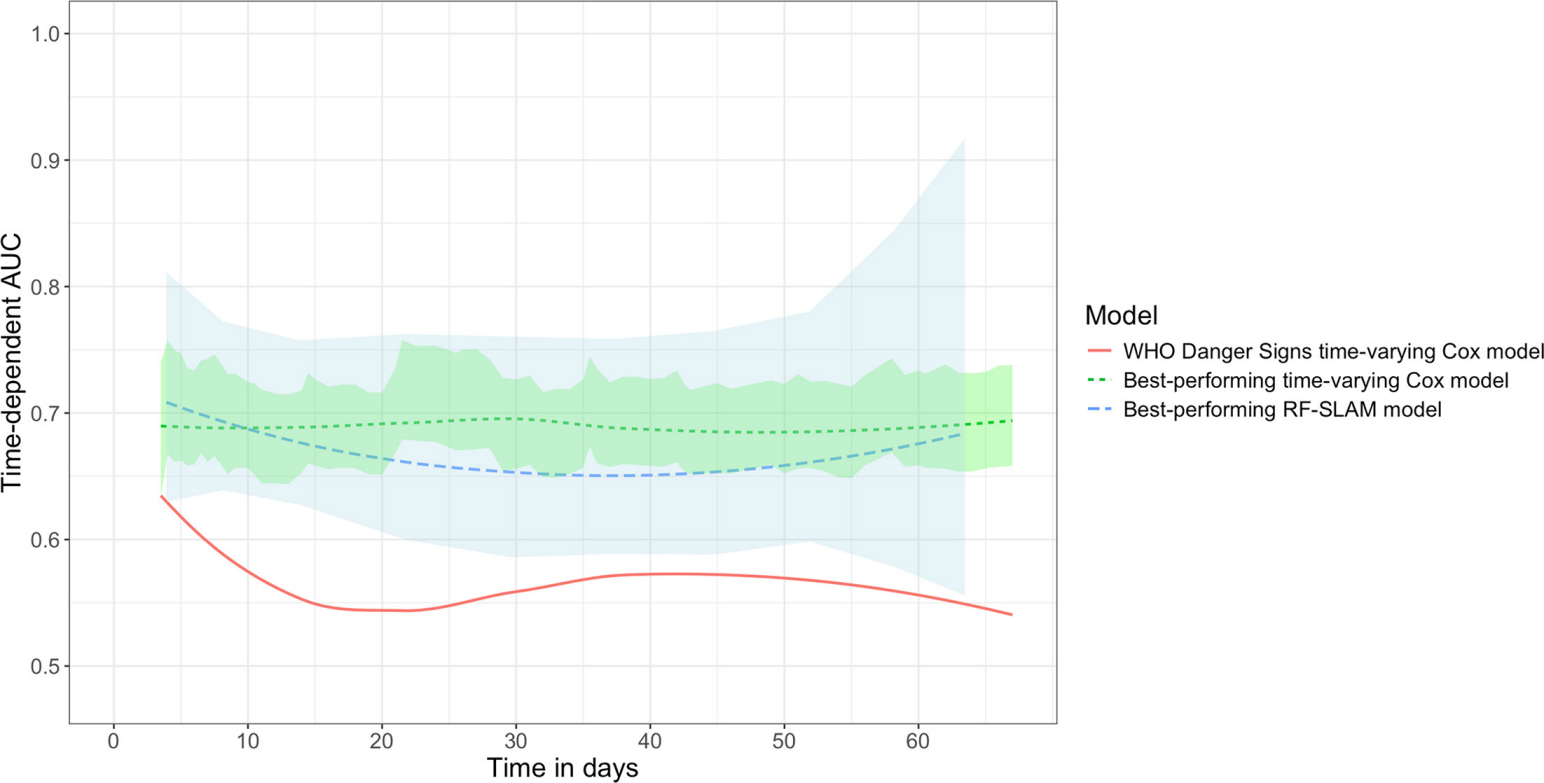
Time-dependent area under the receiver operating characteristic curve (AUC) for best-performing time-varying Cox model for hospitalisation and/or death among young infants (using prevalence of predictors ≥1%), time-varying Cox model based on WHO danger signs and best-performing RF-SLAM model. Note: Green and blue shaded areas represent 95% CIs. The 95% CI for the WHO danger signs time-varying Cox model (red) is not shown because the CI was very wide and not useful for illustrative purposes. AUC, area under the receiver operating characteristic curve; RF-SLAM, random forest for survival, longitudinal and multivariate data analysis.

The best-performing multivariable Cox model included seven predictors and had a C-statistic of 0.71 (95% CI 0.68 to 0.75) with a similar cross-validated C-statistic (table 2). The model had a consistent time-dependent AUC across the observation period (figure 2).

**Table 2:** Discrimination of best-performing time-varying Cox model; time-varying Cox model based on WHO danger signs; and best-performing time-varying Cox model limited to clinical features (with additional covariates removed) plus a variable denoting at least one WHO danger sign for hospitalisation and/or death among young infants (using predictors with prevalence ≥1%)

	Predictor	Prevalence, n (%)*	Adjusted HR (95% CI)	P value	C-statistic (95% CI)	Cross-validated C-statistic (95% CI)
Best-performing time-varying Cox model						
Additional covariates	Any perinatal or delivery complication	864 (5.4)	1.3 (0.71 to 2.2)	0.43	0.71 (0.68 to 0.75)	0.68 (0.58 to 0.78)
	Umbilical cord care					
	Antiseptic	9546 (60)	1.6 (0.92 to 2.8)	0.096		
	Antiseptic and other substance(s)	3241 (20)	1.5 (0.79 to 2.8)	0.23		
	Other substance(s)	941 (5.9)	1.6 (0.71 to 3.5)	0.26		
	Gestational age at birth (weeks), median (25th, 75th)†	39.1 (38.3, 40.1)	1.1 (1.0 to 1.2)	0.032		
History	Stuffy nose	1142 (7.1)	3.0 (2.0 to 4.6)	<0.001		
	Yellow discolouration of skin or eyes	199 (1.2)	11 (6.6 to 17)	<0.001		
	Unusual skin rash or anything abnormal on skin	183 (1.1)	2.3 (1.1 to 4.8)	0.024		
Physical examination	Cough	178 (1.1)	7.5 (4.4 to 13)	<0.001		
Time-varying Cox model based on WHO danger signs						
History	Abnormal movement (convulsions/fits)	9 (0.056)	15 (2.1 to 107)	0.0073	0.56 (0.54 to 0.60)	0.51 (0.47 to 0.55)
Physical examination	Not sucking effectively	17 (0.11)	14 (4.2 to 46)	<0.001		
	Fast breathing (RR ≥60 per min)	19 (0.12)	2.3 (0.48 to 12)	0.30		
	Severe lower chest wall in-drawing	14 (0.087)	20 (5.0 to 81)	<0.001		
	Lethargy (no movement or movement only on stimulation)	4 (0.025)	48 (8.4 to 275)	<0.001		
	Fever (one temperature measurement ≥38°C or two measurements ≥37.5°C)‡	11 (0.069)	32 (9.9 to 102)	<0.001		
	Low body temperature (one temperature measurement<34.5°C or two measurements<35.5°C)‡	7 (0.047)	2.4 (0.13 to 43)	0.56		
	Yellow discolouration of hands and soles of feet	13 (0.081)	31 (12 to 78)	<0.001		
Physical examination	1 of any of the 8 danger signs	83 (0.52)	20 (12 to 33)	<0.001	0.56 (0.53 to 0.60)	0.54 (0.49 to 0.58)
Best-performing time-varying Cox model limited to clinical features (with additional covariates removed) plus a variable denoting at least one WHO danger sign						
History	Stuffy nose	1142 (7.1)	3.0 (2.0 to 4.5)	<0.001	0.70 (0.67 to 0.74)	0.68 (0.59 to 0.76)
	Yellow discolouration of skin or eyes	199 (1.2)	8.9 (5.5 to 14)	<0.001		
	Unusual skin rash or anything abnormal on skin	183 (1.1)	2.9 (1.4 to 5.9)	0.0044		
Physical examination	Cough	178 (1.1)	5.6 (3.1 to 10)	<0.001		
	1 of any of the 8 WHO danger signs	83 (0.52)	6.0 (3.4 to 11)	<0.001		

The cumulative sum of visits with red, oozing and/or swollen eyes up to the last visit prior to the current visit met the criteria to be selected as a candidate predictor and was included after backward selection (online supplemental table S6). Since this predictor was the only aggregative time-varying operationalisation in the final set of candidate predictors, its removal would support the feasibility of the final best-performing time-varying Cox model. Removing this predictor changed the C-statistic of the best-performing time-varying Cox model minimally from 0.72 (95% CI 0.69 to 0.76) to 0.71 (95% CI 0.68 to 0.75) and it was therefore removed to achieve a more parsimonious and feasible final multivariable model.

* Denominator is total number of in-person visits (n=16 023).

† Adjusted HR inverted so that lower gestational age corresponds to higher risk.

‡ Thresholds for fever and low body temperature vary slightly across WHO guidelines.

RR, respiratory rate.

The WHO eight danger signs were infrequent (<0.1% of visits). A time-varying Cox model based on WHO danger signs or a single variable denoting at least one of these signs both had a C-statistic of 0.56 (95% CI 0.54 to 0.60) with a similar cross-validated C-statistic (table 2). The time-dependent AUC value of the WHO danger signs was higher during the first week of age and subsequently decreased (figure 2).

The best-performing time-varying Cox model with the addition of a variable denoting at least one WHO danger sign had a C-statistic of 0.73 (95% CI 0.69 to 0.76) (online supplemental table S8). A time-varying Cox model consisting of the four clinical features from the best-performing Cox model plus a variable denoting at least one WHO danger sign had a C-statistic of 0.70 (95% CI 0.67 to 0.74) (table 2).

In an additional analysis limiting predictors to baseline additional covariates and caregiver-reported symptoms on history alone during in-person visits, the best-performing time-varying Cox model discrimination was similar (online supplemental table S9). When the additional covariates in the best-performing time-varying Cox model were removed, leaving only four clinical features, the C-statistic decreased slightly (online supplemental table S10).

Other additional sensitivity and subgroup analyses demonstrated no substantial changes in discrimination (online supplemental tables S11–S14).

The best-performing time-varying Cox model (table 2) was derived using data from up to 11 CHRW visits. As the maximum number of CHRW home visits per infant decreases, thereby increasing the average prediction window (ie, number of days between an event and the most recent visit), the C-statistic decreases (online supplemental figure S3).

**Figure 3:**
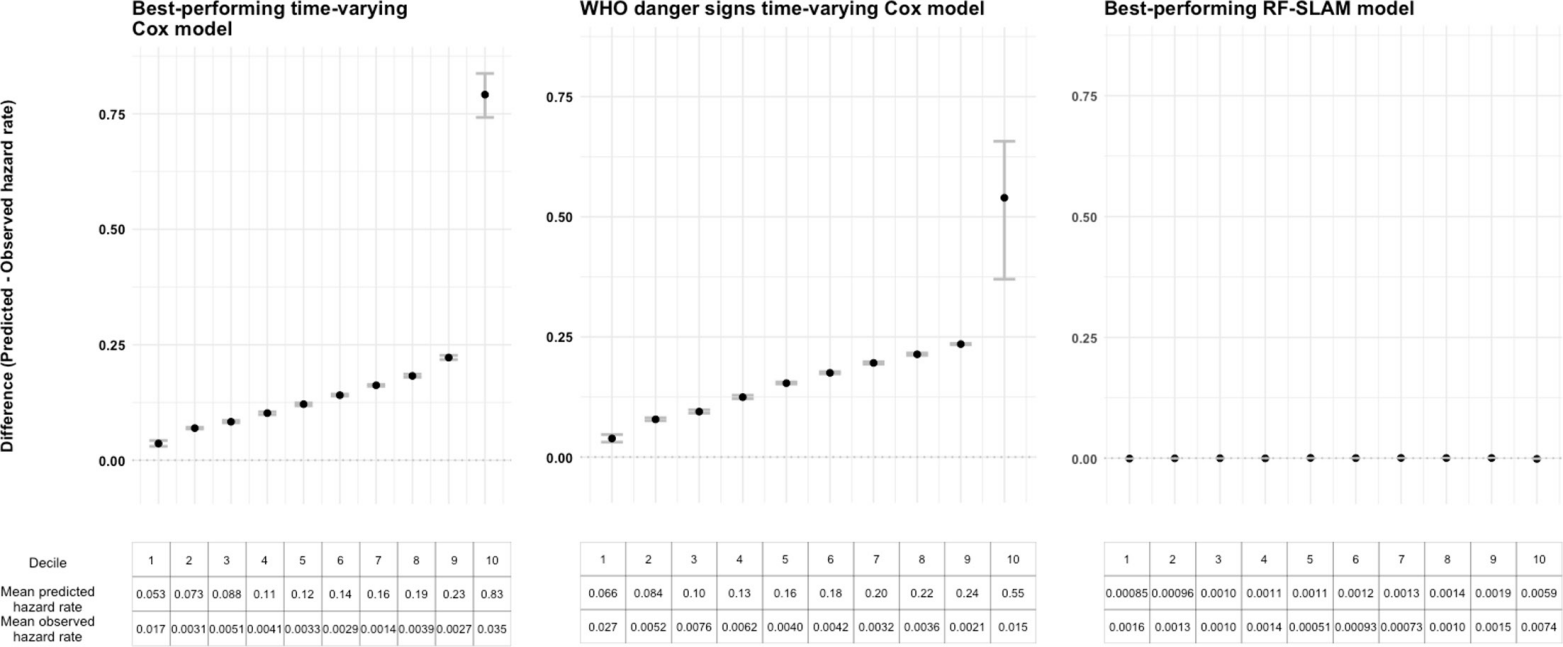
Calibration plots by decile of predicted hazard rates for best-performing time-varying Cox model, time-varying Cox model based on WHO danger signs and best-performing RF-SLAM model. Discrete risk groups were defined by deciles of the predicted hazard rates with the first decile having the lowest predicted hazard rates and the 10th decile having the highest predicted hazard rates. Calibration was assessed by risk group. For each decile risk group, the mean predicted hazard rate was compared with the mean observed hazard rate. The difference between the mean predicted and observed hazard rates was computed and then plotted for each corresponding decile. The grey bars indicate the 95% CIs from 1000 bootstrapped data sets. For well-calibrated models, the difference between the predicted and observed hazard rates should be minimal (near 0). RF-SLAM, random forest for survival, longitudinal and multivariate data analysis.^23^

Across all deciles of predicted risks, both the best-performing time-varying Cox model and WHO danger signs Cox model overestimated predicted risks with gradually increasing differences between predicted and observed hazard rates and 95% CIs excluding 0 (figure 3).

The 42 predictors in the best-performing RF-SLAM model (p-value<0.2 in unadjusted analysis, VIF<5 and prevalence ≥0.1%), which included both visit-specific and aggregative predictors, are shown in online supplemental figure S4. Variables with predictive importance are those with a minimal depth less than the depth threshold of 10.99. The best-performing RF-SLAM model had a C-statistic of 0.69 (95% CI 0.64 to 0.73) and was well calibrated (figure 3). The RF-SLAM model had a higher AUC at the start and end of the observation period (figure 2).

A RF-SLAM model using predictors with a prevalence of ≥1%, instead of ≥0.1% (13 predictors, all baseline covariates or visit-specific clinical features), had a C-statistic of 0.64 (95% CI 0.61 to 0.69) (online supplemental figure S5).

A RF-SLAM model using the seven predictors (three baseline covariates and four visit-specific clinical features) included in the best-performing time-varying Cox model had a C-statistic of 0.66 (95% CI 0.63 to 0.72) (online supplemental figure S6).

## Discussion

A Cox model using time-fixed baseline covariates and visit-specific time-varying clinical features demonstrated higher discrimination and similar calibration compared with the conventional approach using WHO danger signs to predict hospitalisation and/or death among young infants born generally healthy and assessed during CHW routine home visits in Dhaka, Bangladesh. Discrimination was maintained using four clinical features from the best-performing Cox model (nasal congestion, jaundice, skin rash and cough) plus a variable denoting at least one WHO danger sign. Aggregative time-varying operationalisations of predictors had weaker associations with hospitalisation and/or death than visit-specific operationalisations, were not retained in the best-performing Cox model and did not add substantial discriminatory value in the best-performing RF-SLAM model. The best-performing RF-SLAM model was well calibrated with similar discrimination to the best-performing time-varying Cox model.

In a 2024 systematic review of clinical sign-based algorithms to identify sepsis in young infants,^11^ the most accurate algorithm in the routine home visit setting was the WHO danger signs. Although WHO danger signs had poorer discrimination than the best-performing Cox model in the present secondary analysis of the SEPSiS cohort, they remained important given their high specificity (~95%) for severe illness requiring referral^10^ and significant association with mortality.^8^ However, the frequency of WHO danger signs in the SEPSiS cohort was low (<0.1% of visits) compared with prior large studies of young infants in South Asia (ANISA)^25^ and sub-Saharan Africa (AFRINEST).^26 27^ Compared with these cohorts, which included higher-risk infants, rural populations and fewer exclusion criteria, the SEPSiS population was relatively lower-risk, urban and facility-recruited, likely contributing to the lower frequency and poorer predictive performance of the WHO danger signs observed in our analysis.

Clinical features in the best-performing Cox model, including nasal congestion, jaundice, skin rash and cough, were more frequently ascertained during routine visits than WHO danger signs. Pneumonia accounted for a substantial proportion of hospitalisations, many of which were likely viral in aetiology; in these cases, milder and more common symptoms such as nasal congestion and cough may have higher predictive accuracy than WHO danger signs.

While WHO danger signs remain essential for identifying severe illness during routine visits, our findings suggest that population risk and setting may affect their frequency and performance, and that they may not capture the full spectrum of infants requiring hospitalisation in lower-risk, urban populations.

The SEPSiS observational cohort study was conducted during the COVID-19 pandemic. In young infants, SARS-CoV-2 infection is generally mild,^28 29^ suggesting minimal direct impact on study outcomes. The pandemic may have had indirect effects on hospitalisation rates in both directions, through delayed care-seeking during lockdown periods,^30^ potentially leading to higher severity at presentation and increased hospitalisations or decreased exposure to infectious pathogens, which may have led to fewer hospitalisations. Telephone visits, which occurred more frequently during periods with stricter COVID-19 restrictions (online supplemental figure S7), were excluded from this analysis. However, over 80% of visits were conducted in person, likely limiting any impact on the findings. Overall, the pandemic is unlikely to have introduced systematic bias in the associations or predictive performance observed.

We hypothesised that aggregative time-varying predictors would have higher predictive accuracy than their corresponding visit-specific operationalisations. From a clinical standpoint, cumulative days or weeks of symptoms would be more concerning than symptoms at a single visit. However, despite up to 11 scheduled visits, this frequency may not have been sufficient to capture the rapid progression of severe illnesses requiring hospitalisation in young infants. For example, conditions such as sepsis can evolve over hours to days, making it unlikely that aggregating data across visits spaced four to 7 days apart would provide additional benefit beyond information from the most recent visit alone.

We further hypothesised that a machine learning model such as RF-SLAM would outperform the time-varying Cox model. However, even when including a larger set of predictors, and despite the theoretical advantages of random forest over traditional regression (online supplemental panel S4), the best-performing RF-SLAM model had similar discrimination as the best-performing Cox model with better calibration. When the RF-SLAM model was restricted to the seven predictors included in the best-performing Cox model, discrimination was slightly lower, but remained comparable. These findings suggest that using machine learning models such as random forest may not always result in higher discrimination. While some studies have demonstrated that machine learning methods, including random forest, can improve discrimination compared with traditional regression,^13 31^ another study found that regression performed comparably to machine learning models.^32^

A limitation of this study was that the observation period started at the first CHRW home visit (day 3–7 of age), excluding the first 24 hours of age, when neonatal mortality is highest.^33^ However, infants remained at high risk of hospitalisation and death from days 3–67 of age, substantiating the importance of prediction models applicable to this period. Second, prevalence of symptoms and signs during routine home visits was relatively low, likely reflecting the urban setting of Dhaka where health facilities are numerous, caregivers readily seek care for infant illness and CHWs are less likely to ascertain concerning features during scheduled visits. In a separate analysis of severe infection incidence in this cohort, 85% of severe infection episodes were identified following caregiver self-referral compared with 15% identified following a scheduled CHRW home visit assessment and referral.^17^ Third, the baseline additional covariates in the best-performing Cox model may not be readily available outside of a research setting. For example, information regarding gestational age at birth was obtained from a variety of sources including the mother’s antenatal card and ultrasound report. Nevertheless, a sensitivity analysis removing the baseline additional covariates did not substantially reduce discrimination. Fourth, the protocol-defined referral system, mandating referral to a study physician if a CHRW ascertained one of the WHO danger signs during a visit, may have increased the association of the danger signs with hospitalisation, thereby potentially introducing a bias that may improve discrimination of the WHO danger signs model. However, despite this potential protocol-induced bias, the best-performing Cox model did not include any of the WHO danger signs and showed higher discrimination than the WHO danger signs model. Fifth, hospitalisation decisions were made by non-study physicians without standardised protocols. However, this subjectivity would be expected to bias associations towards the null rather than systematically bias associations or predictive performance in a particular direction. In addition, although physicians assessing the outcome were not study staff, there was no protocol-instituted blinding to CHRW assessments, and physician recommendations for hospitalisation could have been influenced by CHRW findings.

### Implications for clinical practice and future research

Our findings should be interpreted in the context of current WHO^34^ and Integrated Management of Childhood Illness (IMCI) guidelines,^6^ which use WHO danger sign-based algorithms to guide referral and treatment decisions. The best-performing Cox model had a C-statistic of 0.71, and even when combined with WHO danger signs (C-statistic=0.73), discrimination may not be sufficient to meaningfully change referral decision-making. However, our findings suggest that incorporating additional clinical features may improve the performance of current WHO danger signs in identifying infants who may require referral. As such, the model should not be considered ready for implementation in its current form and is not intended to replace current WHO guideline-based approaches, but rather to serve as an initial step towards identifying additional clinical features that may improve risk stratification. Further model refinement and external validation in diverse LMIC populations, including higher risk infants and those in rural settings, are needed before evaluating impact on referral decision-making and clinical outcomes.

Findings from this study suggest that similar discrimination may be achieved with fewer CHW visits and without additional baseline covariates, providing important insights for future model development and refinement. Considering prior studies demonstrating higher mortality risk associated with multiple concurrent WHO danger signs,^35 36^ future work may explore whether modelling specific combinations of clinical features (eg, simultaneous presence of fever and poor feeding) improves predictive performance. Finally, backward selection using a threshold p-value is commonly used for predictor selection,^37^ but other methods such as using Akaike Information Criterion and penalised regression^38^,^40^ could be implemented in this context and may be considered in future studies.

## Conclusions

During CHW routine home visit assessments of infants born generally healthy in an urban setting, aggregative clinical features do not improve prediction of hospitalisation and/or death compared with visit-specific clinical features. Despite theoretical advantages of machine learning models such as random forest, a time-varying Cox model consisting of three baseline covariates and four visit-specific clinical features had similar discrimination and would be more feasible in clinical practice. Among infants born generally healthy, adding four clinical features—nasal congestion, jaundice, skin rash and cough—to the WHO danger signs algorithm may improve its predictive performance by capturing a broader spectrum of illnesses requiring hospitalisation.

## Supplementary material

10.1136/bmjpo-2026-004626 online supplemental file 1

## Data Availability

Data are available in a public, open access repository.
