# Supplementary material for: Development of a prediction model for infant hospitalisation and death using clinical features assessed by community health workers during routine postnatal home visits in Dhaka, Bangladesh
Source: BMJ Paediatr Open. 2026 Jun 25;10(1):e004626. doi: 10.1136/bmjpo-2026-004626 (PMC13311572; doi:10.1136/bmjpo-2026-004626)
Supplement: Supplementary file 2 [file bmjpo-2026-004626.reviewer_comments.pdf]

PEER REVIEW HISTORY

BMJ Paediatrics Open publishes all reviews undertaken for accepted manuscripts. Reviewers are asked to complete a checklist review form and are provided with free text boxes to elaborate on their assessment. These free text comments are reproduced below.

ARTICLE DETAILS

**TITLE (PROVISIONAL)**	Development of a prediction model for infant hospitalization and death using clinical features assessed by community health workers during routine postnatal home visits in Dhaka, Bangladesh
**AUTHORS**	Fung, Alastair<br>Sappani, Marimuthu<br>Heasley, Cole<br>Chen, Chun-Yuan<br>Morris, Shaun<br>Gill, Peter<br>Bassani, Diego G<br>Hamer, Davidson H.<br>Shah, Prakesh<br>Gaffar, S. M. Abdul<br>Yeasmin, Sultana<br>Sarker, Shafiqul<br>Sultana, Shamima<br>Beyene, Joseph<br>Roth, Daniel E.

VERSION 1 – REVIEW

**REVIEWER NAME**	Venzor Strader, Anahí
**REVIEWER AFFILIATION**	Boston Children's Hospital, Emergency MEdicine
**REVIEWER CONFLICT OF INTEREST**	No Conflict of Interest
**AI DISCLOSURE**	No, I have not used AI
**DATE REVIEW RETURNED**	25-Mar-2026

**GENERAL COMMENTS**	I appreciate the opportunity to review this manuscript, which makes valuable contributions to the field. The major strengths of this work include the appropriate use of a time-varying Cox model and its comparison to a machine learning–based random forest approach. It also contributes meaningfully to the literature by evaluating the risk of hospitalization and death among infants assessed through home visits, rather than at health facilities, thereby capturing a generally healthier cohort of newborns.<br><br>My comments for review:<br>- It is concerning that the model relies on data-driven variable selection (unadjusted p-values <0.2) rather than pre-specification of predictors or the use of penalized regression methods. This approach may introduce optimism bias. Did the authors explore alternative strategies for model building, and how might this choice impact the robustness of the results?<br>- Missing data are addressed only briefly. More detail on the pattern and mechanism of missingness would be helpful to better understand its potential impact on the findings.<br>- Hospitalization criteria can vary significantly across hospitals and

	even between providers, particularly for the most common conditions reported (e.g., pneumonia and jaundice). Providing additional context on inter-hospital and inter-provider variability would help better frame the outcome. How standardized are admission protocols and criteria? To what extent might this subjectivity affect the performance and predictive power of the models?<br>- The clinical utility of the model remains somewhat unclear. While the reported discrimination is moderate, it would be helpful to better understand how this level of performance would translate into real-world decision-making, particularly for CHWs. Would the addition of these predictors meaningfully change referral decisions compared to existing approaches?

**REVIEWER NAME**	Nisar, Yasir Bin
**REVIEWER AFFILIATION**	WHO, MCA
**REVIEWER CONFLICT OF INTEREST**	No Conflict of Interest
**AI DISCLOSURE**	Grammarly
**DATE REVIEW RETURNED**	06-Apr-2026

**GENERAL COMMENTS**	This secondary analysis of the SEPSiS cohort conducted in urban Dhaka, Bangladesh, assessed models for improving the detection of young infant hospitalization or death risk during routine community health worker (CHW) visits. Utilizing clinical features and covariates, the optimal Cox model, which included four visit-specific features and three baseline covariates, demonstrated superior performance compared to the WHO danger signs algorithm (C-statistic 0.71 vs. 0.56), with comparable calibration. Incorporation of WHO danger signs into the model yielded similar outcomes, while more complex approaches did not enhance predictive accuracy. The study adhered to TRIPOD+AI reporting standards, involved sensitivity analyses, and identified key limitations.<br>Prior to consideration for publication, minor revisions are required as detailed below:<br>1. The authors defined fast breathing as a respiratory rate >60 breaths per minute. In contrast, the WHO defines fast breathing as 60 or more breaths per minute. Kindly elaborate on the rationale for adopting a different definition.<br>2. The statement “A total of 1906 infants had at least one CHRW home visit and were included” requires clarification: Why was only one visit considered? How was outcome data (hospitalization or death) obtained when only a single visit occurred?<br>3. With regard to the notation “CHRWs conducted 20,472 visits (82% in-person, 18% by telephone),” please clarify how CHRWs recorded clinical signs such as temperature, respiratory rate, feeding, and movement during telephone consultations, given the substantial proportion (18%) conducted remotely.<br>4. The manuscript references “systematic antibiotic administration” but lacks specific details. Who received antibiotics, in which settings (outpatient or inpatient), and what was the duration of treatment? Please provide further information.<br>5. As pneumonia accounted for the highest proportion of hospitalizations (42%), please specify the definition of pneumonia used. Notably, the WHO recommends outpatient management for young infants aged 7–59 days presenting solely with fast breathing (pneumonia), without need for hospitalization.<br>6. Please clarify whether clinical features were assessed individually

	or in combination, as previous studies have reported higher mortality associated with multiple signs (Puri et al., Nisar et al.).<br>7. Please discuss your key findings in relation to the current WHO PSBI guidelines (2025), IMNCI protocols (2019), and any ongoing algorithm revision processes.<br>8. Kindly comment on the generalizability of your results, bearing in mind that the study cohort was urban, recruited from facilities, generally healthy due to exclusion of high-risk infants, exhibited high antenatal care coverage (97%), low rates of preterm/low-birth-weight births, and had easy hospital access, in contrast to rural populations described in prior studies (e.g., ANISA, AFRINEST secondary analyses by Puri et al. and Nisar et al.). Please consider including a discussion regarding external validation across diverse LMIC contexts.<br>9. Please address potential subjective bias concerning hospitalization, particularly instances where physicians recommended admission but families declined. Is there outcome data (death or survival) available for these infants?<br>10. Given that the study period was from November 25, 2020, to February 18, 2022, please discuss any possible impact of the COVID-19 pandemic.

VERSION 1 – AUTHOR RESPONSE

Reviewer: 1
Dr. Anahí Venzor Strader, Boston Children's Hospital
Comments to the Author
I appreciate the opportunity to review this manuscript, which makes valuable contributions to the field. The major strengths of this work include the appropriate use of a time-varying Cox model and its comparison to a machine learning–based random forest approach. It also contributes meaningfully to the literature by evaluating the risk of hospitalization and death among infants assessed through home visits, rather than at health facilities, thereby capturing a generally healthier cohort of newborns.

My comments for review:

COMMENT 1 - It is concerning that the model relies on data-driven variable selection (unadjusted p-values <0.2) rather than pre-specification of predictors or the use of penalized regression methods. This approach may introduce optimism bias. Did the authors explore alternative strategies for model building, and how might this choice impact the robustness of the results?

RESPONSE: We thank the reviewer for this important comment. We acknowledge that our modeling approach was partially data-driven, particularly in the variable selection process. However, the 45 clinical features considered as candidate predictors were pre-specified based on clinical relevance. These variables were prospectively collected and their inclusion at the time of study design was informed by clinical input from co-investigators during the development of the CHW home visit assessment questionnaire within the SEPSiS study.

While we acknowledge that backward selection using p-values may lead to overfitting, there is evidence that the performance of variable selection methods, in terms of parsimony and discrimination of the final model, depends on factors such as the size of the dataset. For example, Sanchez-Pinto et al. compared eight different variable selection methods for prediction modeling using two clinical datasets of different sizes (https://pmc.ncbi.nlm.nih.gov/articles/PMC6003624/). They demonstrated that in a smaller clinical dataset (n=6564), backward selection using p-values achieved better parsimony and comparable discrimination compared to penalized regression methods such as Least Absolute Shrinkage and Selection Operator (LASSO) and elastic net. With 1906

infants, our study dataset would be considered small, and thus a backward selection approach using p-values was felt to be reasonable in this context.

In addition, we conducted an analysis in which WHO danger signs were pre-specified and included in the variable selection process regardless of their statistical significance, acknowledging their established clinical importance. This analysis demonstrated similar model performance, supporting the robustness of our findings.

We also performed internal validation using cross-validation to mitigate optimism bias. Cross-validated performance was slightly lower but comparable to the apparent performance, as expected, providing a more conservative estimate of the C-statistic and suggesting limited overfitting.

Furthermore, a sensitivity analysis using a more stringent variable selection threshold of $p<0.05$ yielded similar results (Supplemental Table S14), suggesting that findings were not highly sensitive to the choice of threshold.

Finally, we explored an alternative modeling approach using a random forest model, which demonstrated similar discrimination. Taken together, these analyses suggest that the results were not highly sensitive to reasonable variations in predictor selection and modeling approach.

We have added the following sentences to the Methods section under the subheading “Primary predictors” (page 12, line 20-23) (newly added text is shown in red):

“Primary candidate predictors were 45 clinical features (25 symptoms from caregiver history, 20 physical exam signs) assessed by CHRWs during scheduled home visits. These features were pre-specified based on clinical relevance.”

We have also modified the following sentence at the end of the Discussion as follows (page 28, line 25-32) (newly added text is shown in red):

“Lastly, backward selection using a threshold p-value is commonly used for predictor selection,37 but other methods such as using Akaike Information Criterion (AIC) and penalized regression38-40 could be implemented in this context and may be considered in future studies.”

COMMENT 2 - Missing data are addressed only briefly. More detail on the pattern and mechanism of missingness would be helpful to better understand its potential impact on the findings.

RESPONSE: As stated in the Methods subsection “Sample size and missing data,” missingness of clinical features was minimal (1.2% to 1.4%), and therefore complete case analysis was used in the primary analysis. Given the low proportion of missing data, the potential for bias due to missingness is expected to be limited.
To assess robustness of our findings to bias from missingness, we conducted a sensitivity analysis for the best-performing Cox model using last observation carried forward (LOCF), which yielded a similar C-statistic (Supplemental Table S14), suggesting that missing data did not substantially impact the results.

We have modified the following sentence in the Methods subsection “Sample size and missing data” (page 13, line 18-23) (newly added text is shown in red):

“However, given minimal missingness of clinical features (1.2% to 1.4%), the risk of bias due to missing data was considered low, and models were developed using complete case analysis.”

COMMENT 3 - Hospitalization criteria can vary significantly across hospitals and even between providers, particularly for the most common conditions reported (e.g., pneumonia and jaundice). Providing additional context on inter-hospital and inter-provider variability would help better frame the outcome. How standardized are admission protocols and criteria? To what extent might this subjectivity affect the performance and predictive power of the models?

RESPONSE: Hospitalization decisions in this study were made by non-study physicians as part of routine clinical care, and there were no standardized study-specific admission protocols. As such, the outcome reflects real-world clinical decision-making and may vary across providers and settings, particularly for conditions such as pneumonia and jaundice.

We agree that hospitalization is an inherently subjective outcome and captures a broad range of underlying pathologies and illness severities. However, this subjectivity and heterogeneity would be expected to bias associations with predictors toward the null rather than systematically bias associations or predictive performance in a particular direction.

We have added the following sentences to the Discussion (page 27, line 19-34) to acknowledge these considerations (text in red has been newly added or modified):

“Fifth, hospitalization decisions were made by non-study physicians without standardized protocols. However, this subjectivity would be expected to bias associations towards the null rather than systematically bias associations or predictive performance in a particular direction. In addition, although physicians assessing the outcome were not study staff, there was no protocol-instituted blinding to CHRW assessments, and physician recommendations for hospitalization could have been influenced by CHRW findings.”

COMMENT 4 - The clinical utility of the model remains somewhat unclear. While the reported discrimination is moderate, it would be helpful to better understand how this level of performance would translate into real-world decision-making, particularly for CHWs. Would the addition of these predictors meaningfully change referral decisions compared to existing approaches?

RESPONSE: We agree that the clinical utility of the model was not sufficiently clear in the original version of the Discussion subsection titled “Implications for clinical practice and future research.” We have revised this subsection to more clearly communicate two key points. First, we emphasize that the best-performing Cox model is not ready for implementation in its current form and should instead be viewed as an initial step toward identifying additional clinical features that may be added to WHO danger signs to improve risk stratification. Second, we clarify the implications for future research, highlighting the need for model refinement and external validation, as well as the importance of prioritizing feasibility in CHW settings, including limiting the number of home visits and reducing reliance on baseline variables that may be difficult to ascertain in LMIC contexts. These revisions aim to better contextualize the findings in terms of both their current limitations and their potential to inform future model development and evaluation.

We have revised the Discussion subsection “Implications for clinical practice and future research” as follows (page 27, line 38 to page 28, line 32) (modified text is shown in red):

“Our findings should be interpreted in the context of current WHO34 and Integrated Management of Childhood Illness (IMCI) guidelines,6 which use WHO danger sign-based algorithms to guide referral and treatment decisions. The best-performing Cox model had a C-statistic of 0.71, and even when

combined with WHO danger signs (C-statistic=0.73), discrimination may not be sufficient to meaningfully change referral decision-making. However, our findings suggest that incorporating additional clinical features may improve the performance of current WHO danger signs in identifying infants who may require referral. As such, the model should not be considered ready for implementation in its current form and is not intended to replace current WHO guideline-based approaches, but rather to serve as an initial step toward identifying additional clinical features that may improve risk stratification. Further model refinement and external validation in diverse LMIC populations, including higher-risk infants and those in rural settings, are needed before evaluating impact on referral decision-making and clinical outcomes.

Findings from this study suggest that similar discrimination may be achieved with fewer CHW visits and without additional baseline covariates, providing important insights for future model development and refinement. Considering prior studies demonstrating higher mortality risk associated with multiple concurrent WHO danger signs,35, 36 (e.g., simultaneous presence of fever and poor feeding) future work may explore whether modeling specific combinations of clinical features (e.g., simultaneous presence of fever and poor feeding) improves predictive performance. Lastly, backward selection using a threshold p-value is commonly used for predictor selection,37 but other methods such as using Akaike Information Criterion (AIC) and penalized regression38-40 could be implemented in this context and may be considered in future studies."

Reviewer: 2
Dr. Yasir Bin Nisar, WHO
Comments to the Author
This secondary analysis of the SEPSiS cohort conducted in urban Dhaka, Bangladesh, assessed models for improving the detection of young infant hospitalization or death risk during routine community health worker (CHW) visits. Utilizing clinical features and covariates, the optimal Cox model, which included four visit-specific features and three baseline covariates, demonstrated superior performance compared to the WHO danger signs algorithm (C-statistic 0.71 vs. 0.56), with comparable calibration. Incorporation of WHO danger signs into the model yielded similar outcomes, while more complex approaches did not enhance predictive accuracy. The study adhered to TRIPOD+AI reporting standards, involved sensitivity analyses, and identified key limitations.
Prior to consideration for publication, minor revisions are required as detailed below:

COMMENT 1. The authors defined fast breathing as a respiratory rate >60 breaths per minute. In contrast, the WHO defines fast breathing as 60 or more breaths per minute. Kindly elaborate on the rationale for adopting a different definition.

RESPONSE: We thank the reviewer for identifying this important point. The instances in which ">60 breaths per minute" was used to define tachypnea/fast breathing in the Introduction and Table 2 were typographical errors. All analyses were conducted using the WHO definition of ≥60 breaths per minute. We have corrected this error throughout the manuscript and tables.

COMMENT 2. The statement "A total of 1906 infants had at least one CHRW home visit and were included" requires clarification: Why was only one visit considered? How was outcome data (hospitalization or death) obtained when only a single visit occurred?

RESPONSE: Infants were included if they had at least one CHRW home visit, as this allowed for collection of predictor data and entry into the cohort for this analysis. Outcome ascertainment did not rely solely on caregiver report at follow-up visits.

We agree that for infants with only a single visit, subsequent hospitalizations could not be identified through caregiver recall at later visits. However, hospitalization was also identified through multiple additional pathways, including identification by study medical officers at study hospitals and protocolized mechanisms to capture admissions to non-study hospitals, whereby caregivers or facilities notified study medical officers. Once a hospitalization was identified, study medical officers collected clinical data related to the hospitalization, prospectively for admissions to study hospitals and retrospectively, as soon as possible, for admissions to non-study hospitals.

Deaths were captured as serious adverse events, either prospectively if death occurred during an admission to a study hospital or retrospectively via caregiver report at later visits or notification mechanisms if death occurred elsewhere.

We have added the following sentences to the “Outcome” subsection of the Methods (page 11, line 47 to page 12, line 13) (newly added text is shown in red):

“Hospitalizations were identified through multiple pathways, including identification by study medical officers for admissions to study hospitals, notification mechanisms for admissions to non-study hospitals, and caregiver report at scheduled visits. Study medical officers assigned diagnostic labels based on the treating (non-study) physician’s diagnosis using predefined categories, with free-text entry permitted when needed. Deaths were captured as serious adverse events, either prospectively if death occurred during an admission to a study hospital or retrospectively via caregiver report at later visits or notification mechanisms if death occurred elsewhere. Verbal autopsy was performed by study physicians for all infant deaths and medically certified causes of death were obtained when available.”

COMMENT 3. With regard to the notation “CHRWs conducted 20,472 visits (82% in-person, 18% by telephone),” please clarify how CHRWs recorded clinical signs such as temperature, respiratory rate, feeding, and movement during telephone consultations, given the substantial proportion (18%) conducted remotely.

RESPONSE: Analyses only included clinical features from in-person CHRW visits. Clinical features from telephone visits were not used for predictor ascertainment, as physical exam signs such as temperature and respiratory rate could not be reliably measured remotely.

We have added the following sentence to the “Primary predictors” subsection of the Methods (page 12, line 27-30) (newly added text is shown in red):

“Predictor ascertainment was restricted to in-person visits, as physical exam signs could not be reliably assessed during telephone visits.”

COMMENT 4. The manuscript references “systematic antibiotic administration” but lacks specific details. Who received antibiotics, in which settings (outpatient or inpatient), and what was the duration of treatment? Please provide further information.

RESPONSE: As part of the standard operating procedures for each scheduled visit, CHRWs asked caregivers whether any medications had been administered to the infant since the last visit, including antibiotic use. For each medication, CHRWs recorded the name, location of administration (home, outpatient clinic, private office, hospital, or other), duration (in days), and route of administration. This included systemic antibiotics (oral or injectable).

We have added the following sentences to Supplemental Panel S2, titled, “Ascertainment and derivation of additional covariates by study personnel” (Supplemental File 1, page 87, line 26-31) (newly added text is shown in red):

“Systemic antibiotic administration refers to caregiver-reported use of oral or injectable antibiotics since the last study visit, including medications administered in home, outpatient, or inpatient settings. At each visit, CHRWs recorded medication name, location of administration, duration (days), and route of administration.”

COMMENT 5. As pneumonia accounted for the highest proportion of hospitalizations (42%), please specify the definition of pneumonia used. Notably, the WHO recommends outpatient management for young infants aged 7–59 days presenting solely with fast breathing (pneumonia), without need for hospitalization.

RESPONSE: We acknowledge that WHO guidelines recommend outpatient management for young infants aged 7-59 days presenting solely with fast breathing. In this study, pneumonia and all admission diagnoses were not defined using strict criteria. Rather, diagnoses were assigned by treating (non-study) physicians as part of routine clinical care at the time of hospitalization. Study medical officers then assigned diagnostic labels based on the treating physician’s diagnosis using predefined categories, with free-text entry permitted when needed. As such, pneumonia diagnoses reflect admission diagnoses documented at the time of or shortly after hospitalization, rather than standardized study-defined criteria. The outcome of interest in this analysis was hospitalization and/or death, and pneumonia was reported descriptively as the most common admission diagnosis.

We have added the following sentence to the “Outcome” subsection of the Methods (within the added text inserted to address Reviewer 2, Comment 2) (page 11, line 52 to page 12, line 4) (newly added text is shown in red):

“Study medical officers assigned diagnostic labels based on the treating (non-study) physician’s diagnosis using predefined categories, with free-text entry permitted when needed.”

COMMENT 6. Please clarify whether clinical features were assessed individually or in combination, as previous studies have reported higher mortality associated with multiple signs (Puri et al., Nisar et al.).

RESPONSE: We agree that it is relevant to acknowledge that prior studies have demonstrated higher mortality associated with the presence of multiple clinical signs, including combinations of signs assessed concurrently (Puri et al. 2021, Nisar et al. 2021). In our study, clinical features were incorporated simultaneously within multivariable prediction models, allowing the model to account for the combined contribution of multiple signs to predict the outcome. However, we did not explicitly evaluate predefined combinations of specific signs (e.g., simultaneous presence of fever and poor feeding) as predictors. Future work may explore whether modeling specific combinations of clinical features improves predictive performance.

We have added the following sentence to the “Implications for clinical practice and future research” subsection in the Discussion, citing the work by Puri et al. 2021 and Nisar et al. 2021 (page 28, line 18-25) (newly added text is shown in red):

“Considering prior studies demonstrating higher mortality risk associated with multiple concurrent WHO danger signs,35, 36 future work may also explore whether modeling specific combinations of clinical features (e.g., simultaneous presence of fever and poor feeding) improves predictive performance.”

COMMENT 7. Please discuss your key findings in relation to the current WHO PSBI guidelines (2025), IMNCI protocols (2019), and any ongoing algorithm revision processes.

RESPONSE: We appreciate the recommendation to include an interpretation of our findings in relation to current WHO recommendations for management of serious bacterial infections in young infants and IMCI protocols. As noted in our response to Reviewer 1, Comment 4, we have revised the Discussion subsection “Implications for clinical practice and future research” to more explicitly situate our findings within the WHO frameworks. We clarify that our model is not intended to replace existing WHO guideline-based approaches, but rather to serve as an initial step toward identifying additional clinical features that may improve risk stratification.

We have revised the first paragraph of the Discussion subsection “Implications for clinical practice and future research” as follows (page 27, line 38 to page 28, line 11) (modified text is shown in red):

“Our findings should be interpreted in the context of current WHO34 and Integrated Management of Childhood Illness (IMCI) guidelines,6 which use WHO danger sign-based algorithms to guide referral and treatment decisions. The best-performing Cox model had a C-statistic of 0.71, and even when combined with WHO danger signs (C-statistic=0.73), discrimination may not be sufficient to meaningfully change referral decision-making. However, our findings suggest that incorporating additional clinical features may improve the performance of current WHO danger signs in identifying infants who may require referral. As such, the model should not be considered ready for implementation in its current form and is not intended to replace current WHO guideline-based approaches, but rather to serve as an initial step toward identifying additional clinical features that may improve risk stratification. Further model refinement and external validation in diverse LMIC populations, including higher-risk infants and those in rural settings, are needed before evaluating impact on referral decision-making and clinical outcomes.”

COMMENT 8. Kindly comment on the generalizability of your results, bearing in mind that the study cohort was urban, recruited from facilities, generally healthy due to exclusion of high-risk infants, exhibited high antenatal care coverage (97%), low rates of preterm/low-birth-weight births, and had easy hospital access, in contrast to rural populations described in prior studies (e.g., ANISA, AFRINEST secondary analyses by Puri et al. and Nisar et al.). Please consider including a discussion regarding external validation across diverse LMIC contexts.

RESPONSE: We agree that the generalizability of our findings is influenced by the characteristics of the SEPSiS cohort, which represents a relatively lower-risk, urban, and facility-recruited population with high antenatal coverage and lower rates of preterm birth and low birthweight compared to the ANISA cohort in South Asia and the AFRINEST cohort in sub-Saharan Africa. We have revised the Discussion to more explicitly acknowledge these differences and to contrast our cohort with the ANISA and AFRINEST cohorts, which included rural populations with fewer exclusion criteria. We now emphasize that these differences in inclusion criteria, baseline risk, and study setting likely influence both the frequency and performance of WHO danger signs in our analysis. We have also added a discussion of generalizability, noting that our findings may not be directly applicable to other LMIC settings, particularly higher-risk and rural populations, and that external validation in diverse contexts is essential before evaluating impact on referral decision-making and clinical outcomes.

We have revised the first sentence of the first paragraph of the Discussion as follows (page 23, line 44-54) (modified text is shown in red):

“A Cox model using time-fixed baseline covariates and visit-specific time-varying clinical features demonstrated higher discrimination and similar calibration compared to the conventional approach using WHO danger signs to predict hospitalization and/or death among young infants born generally healthy and assessed during CHRW routine home visits in Dhaka, Bangladesh.”

We have revised paragraphs 2-4 of the Discussion as follows (page 24, line 15 to page 25, line 11) (modified text is shown in red):

“In a 2024 systematic review of clinical sign-based algorithms to identify sepsis in young infants,11 the most accurate algorithm in the routine home visit setting was the WHO danger signs. Although WHO danger signs had poorer discrimination than the best-performing Cox model in the present secondary analysis of the SEPSiS cohort, they remained important given their high specificity (~95%) for severe illness requiring referral10 and significant association with mortality.8 However, the frequency of WHO danger signs in the SEPSiS cohort was low (<0.1% of visits) compared with prior large studies of young infants in South Asia (ANISA)25 and sub-Saharan Africa (AFRINEST).26, 27 Compared to these cohorts, which included higher-risk infants, rural populations, and fewer exclusion criteria, the SEPSiS population was relatively lower-risk, urban, and facility-recruited, likely contributing to the lower frequency and poorer predictive performance of the WHO danger signs observed in our analysis.

Clinical features in the best-performing Cox model, including nasal congestion, jaundice, skin rash, and cough, were more frequently ascertained during routine visits than WHO danger signs. Pneumonia accounted for a substantial proportion of hospitalizations, many of which were likely viral in etiology; in these cases, milder and more common symptoms such as nasal congestion and cough may have higher predictive accuracy than WHO danger signs.

While WHO danger signs remain essential for identifying severe illness during routine visits, our findings suggest that population risk and setting may affect their frequency and performance, and that they may not capture the full spectrum of infants requiring hospitalization in lower-risk, urban populations.”

As noted in our response to Reviewer 2, Comment 7, the last sentence of the first paragraph of the Discussion subsection “Implications for clinical practice and future research” highlights the importance of external validation across diverse LMIC settings (page 28, line 5-11) (newly added text is shown in red):

“Further model refinement and external validation in diverse LMIC populations, including higher-risk infants and those in rural settings, are needed before evaluating impact on referral decision-making and clinical outcomes.”

COMMENT 9. Please address potential subjective bias concerning hospitalization, particularly instances where physicians recommended admission but families declined. Is there outcome data (death or survival) available for these infants?

RESPONSE: As noted in our response to Reviewer 1, Comment 3, we agree that hospitalization decisions are inherently subjective and may vary across providers and settings. We have clarified this in the Discussion.

As noted in the “Outcome” subsection of the Methods, hospitalizations recommended by physicians but declined by caregivers were included as outcomes and retained in the analysis. These instances were included to avoid under-ascertainment of clinically significant illness. We examined the small

number of cases in which hospitalization was recommended but declined by caregivers (n=9). Outcome data was available for all of these infants: all survived and two were subsequently hospitalized the following day.

We have added the following sentence describing these findings to the first paragraph of the Results section (page 16, line 11-16) (newly added text is shown in red):
“Among nine infants for whom hospitalization was recommended but declined by caregivers, all survived and two were subsequently hospitalized the following day.”

As noted in our response to Reviewer 1, Comment 3, we have added the following sentences to the Discussion to acknowledge the subjectivity of hospitalization decisions (page 27, line 19-34) (text in red has been newly added or modified):

“Fifth, hospitalization decisions were made by non-study physicians without standardized protocols. However, this subjectivity would be expected to bias associations towards the null rather than systematically bias associations or predictive performance in a particular direction. In addition, although physicians assessing the outcome were not study staff, there was no protocol-instituted blinding to CHRW assessments, and physician recommendations for hospitalization could have been influenced by CHRW findings.”

COMMENT 10. Given that the study period was from November 25, 2020, to February 18, 2022, please discuss any possible impact of the COVID-19 pandemic.

RESPONSE: We have revised the Discussion to address the potential impact of the COVID-19 pandemic on our findings, including possible effects on care-seeking behaviour, hospitalization rates, and data collection processes.

We have added the following text to the Discussion (page 25, line 13-35) (newly added text is shown in red):

“The SEPSiS observational cohort study was conducted during the COVID-19 pandemic. In young infants, SARS-CoV-2 infection is generally mild,28, 29 suggesting minimal direct impact on study outcomes. The pandemic may have had indirect effects on hospitalization rates in both directions, through delayed care-seeking during lockdown periods,30 potentially leading to higher severity at presentation and increased hospitalizations, or decreased exposure to infectious pathogens, which may have led to fewer hospitalizations. Telephone visits, which occurred more frequently during periods with stricter COVID-19 restrictions (Supplemental Figure S7), were excluded from this analysis. However, over 80% of visits were conducted in person, likely limiting any impact on the findings. Overall, the pandemic is unlikely to have introduced systematic bias in the associations or predictive performance observed.”

REFERENCES

1. UNICEF and partners in the United Nations Inter-Agency Group for Child Mortality Estimation. Levels and Trends in Child Mortality Report 2025: Estimates developed by the United Nations Inter-agency Group for Child Mortality Estimation: United Nations Children’s Fund; 2026 [Available from: https://data.unicef.org/resources/levels-and-trends-in-child-mortality-2025/.
2. Tiruneh GT, Shiferaw CB, Worku A. Effectiveness and cost-effectiveness of home-based postpartum care on neonatal mortality and exclusive breastfeeding practice in low-and-middle-income countries: a systematic review and meta-analysis. BMC Pregnancy Childbirth. 2019;19(1):507.
3. Lassi ZS, Kedzior SG, Bhutta ZA. Community-based maternal and newborn educational care

packages for improving neonatal health and survival in low- and middle-income countries. Cochrane Database Syst Rev. 2019;2019(11).
4. Rudd KE, Johnson SC, Agesa KM, Shackelford KA, Tsoi D, Kievlan DR, et al. Global, regional, and national sepsis incidence and mortality, 1990-2017: analysis for the Global Burden of Disease Study. Lancet. 2020;395(10219):200-11.
5. Sankar MJ, Natarajan CK, Das RR, Agarwal R, Chandrasekaran A, Paul VK. When do newborns die? A systematic review of timing of overall and cause-specific neonatal deaths in developing countries. J Perinatol. 2016;36 Suppl 1(Suppl 1):S1-s11.
6. Integrated Management of Childhood Illness: management of the sick young infant aged up to 2 months Geneva: World Health Organization; 2019 [Available from: https://www.who.int/publications/i/item/9789241516365.
7. WHO recommendations on maternal and newborn care for a positive postnatal experience Geneva: World Health Organization; 2022 [Available from: https://www.who.int/publications/i/item/9789240045989.
8. Darmstadt GL, Ahmed S, Islam MS, Abdalla S, El Arifeen S, Arvay ML, et al. Association of clinical signs of possible serious bacterial infections identified by community health workers with mortality of young infants in South Asia: a prospective, observational cohort study. EClinicalMedicine. 2025;80:103070.
9. Young Infants Clinical Signs Study Group. Clinical signs that predict severe illness in children under age 2 months: a multicentre study. Lancet. 2008;371(9607):135-42.
10. Darmstadt GL, Baqui AH, Choi Y, Bari S, Rahman SM, Mannan I, et al. Validation of a clinical algorithm to identify neonates with severe illness during routine household visits in rural Bangladesh. Arch Dis Child. 2011;96(12):1140-6.
11. Fung A, Shafiq Y, Driker S, Rees CA, Mediratta RP, Rosenberg R, et al. Diagnostic Accuracy of Clinical Sign Algorithms to Identify Sepsis in Young Infants Aged 0 to 59 Days: A Systematic Review and Meta-analysis. Pediatrics. 2024;154(Suppl 1).
12. Shafiq Y, Fung A, Driker S, Rees CA, Mediratta RP, Rosenberg R, et al. Predictive Accuracy of Infant Clinical Sign Algorithms for Mortality in Young Infants Aged 0 to 59 Days: A Systematic Review. Pediatrics. 2024;154(Suppl 1).
13. Ramgopal S, Horvat CM, Yanamala N, Alpern ER. Machine Learning To Predict Serious Bacterial Infections in Young Febrile Infants. Pediatrics. 2020;146(3).
14. Neal SR, Sturrock SS, Musorowegomo D, Gannon H, Zaman M, Cortina-Borja M, et al. Clinical prediction models to diagnose neonatal sepsis in low-income and middle-income countries: a scoping review. BMJ Glob Health. 2025;10(4).
15. Goldstein BA, Navar AM, Carter RE. Moving beyond regression techniques in cardiovascular risk prediction: applying machine learning to address analytic challenges. Eur Heart J. 2017;38(23):1805-14.
16. SEPSIS Observational Cohort Study in Young Infants in Bangladesh [Available from: https://ClinicalTrials.gov/show/NCT04012190.
17. Fung A, Heasley C, Pell LG, Bassani DG, Shah PS, Morris SK, et al. Severe infection incidence among young infants in Dhaka, Bangladesh: an observational cohort study. BMJ Public Health. 2025;3(2):e002383.
18. Collins GS, Moons KGM, Dhiman P, Riley RD, Beam AL, Van Calster B, et al. TRIPOD+AI statement: updated guidance for reporting clinical prediction models that use regression or machine learning methods. Bmj. 2024;385:e078378.
19. Austin PC, White IR, Lee DS, van Buuren S. Missing Data in Clinical Research: A Tutorial on Multiple Imputation. Can J Cardiol. 2021;37(9):1322-31.
20. Vittinghoff E, Glidden DV, Shiboski SC, McCulloch CE. Regression Methods in Biostatistics. 2nd ed. Boston, MA: Springer; 2012.
21. Lavery MR, Acharya P, Sivo SA, Xu L. Number of predictors and multicollinearity: What are their effects on error and bias in regression? Communications in Statistics - Simulation and Computation. 2019;48(1):27-38.

22. Wongvibulsin S, Wu KC, Zeger SL. Clinical risk prediction with random forests for survival, longitudinal, and multivariate (RF-SLAM) data analysis. BMC Med Res Methodol. 2019;20(1):1.
23. Steyerberg EW. Chapter 15.3: Calibration. Clinical Prediction Models: A Practical Approach to Development, Validating, and Updating. Statistics for Biology and Health. 2nd ed. Switzerland: Springer; 2019.
24. R Core Team. R: A Language and Environment for Statistical Computing Vienna, Austria.: R Foundation for Statistical Computing; 2025 [Available from: https://www.R-project.org/.
25. Saha SK, Schrag SJ, El Arifeen S, Mullany LC, Shahidul Islam M, Shang N, et al. Causes and incidence of community-acquired serious infections among young children in south Asia (ANISA): an observational cohort study. Lancet. 2018;392(10142):145-59.
26. Tshefu A, Lokangaka A, Ngaima S, Engmann C, Esamai F, Gisore P, et al. Oral amoxicillin compared with injectable procaine benzylpenicillin plus gentamicin for treatment of neonates and young infants with fast breathing when referral is not possible: a randomised, open-label, equivalence trial. Lancet. 2015;385(9979):1758-66.
27. Tshefu A, Lokangaka A, Ngaima S, Engmann C, Esamai F, Gisore P, et al. Simplified antibiotic regimens compared with injectable procaine benzylpenicillin plus gentamicin for treatment of neonates and young infants with clinical signs of possible serious bacterial infection when referral is not possible: a randomised, open-label, equivalence trial. Lancet. 2015;385(9979):1767-76.
28. Khoury L, Pillar G, Shehadeh S. COVID-19 in neonates and infants younger than 6 months - a mild viral illness. Eur J Pediatr. 2023;182(7):3287-91.
29. García H, Allende-López A, Morales-Ruíz P, Miranda-Novales G, Villasis-Keever M. COVID-19 in Neonates with Positive RT-PCR Test. Systematic Review. Arch Med Res. 2022;53(3):252-62.
30. Barriers to optimal care and strategies to promote safe and optimal management of sick young infants during the COVID-19 pandemic: A multi-country formative research study. J Glob Health. 2022;12:05023.
31. Cygu S, Seow H, Dushoff J, Bolker BM. Comparing machine learning approaches to incorporate time-varying covariates in predicting cancer survival time. Sci Rep. 2023;13(1):1370.
32. Spooner A, Chen E, Sowmya A, Sachdev P, Kochan NA, Trollor J, et al. A comparison of machine learning methods for survival analysis of high-dimensional clinical data for dementia prediction. Sci Rep. 2020;10(1):20410.
33. Dol J, Hughes B, Bonet M, Dorey R, Dorling J, Grant A, et al. Timing of neonatal mortality and severe morbidity during the postnatal period: a systematic review. JBI Evid Synth. 2023;21(1):98-199.
34. WHO recommendations for management of serious bacterial infections in infants aged 0–59 days Geneva: World Health Organization; 2024 [Available from: https://www.who.int/publications/i/item/9789240102903.
35. Puri D, Nisar YB, Tshefu A, Longombe AL, Esamai F, Marete I, et al. Prevalence of clinical signs of possible serious bacterial infection and mortality associated with them from population-based surveillance of young infants from birth to 2 months of age. PLoS One. 2021;16(2):e0247457.
36. Nisar YB, Tshefu A, Longombe AL, Esamai F, Marete I, Ayede AI, et al. Clinical signs of possible serious infection and associated mortality among young infants presenting at first-level health facilities. PLoS One. 2021;16(6):e0253110.
37. Moons KG, Kengne AP, Woodward M, Royston P, Vergouwe Y, Altman DG, et al. Risk prediction models: I. Development, internal validation, and assessing the incremental value of a new (bio)marker. Heart. 2012;98(9):683-90.
38. Steyerberg EW. Chapter 11: Selection of Main Effects. Clinical Prediction Models: A Practical Approach to Development, Validating, and Updating. Statistics for Biology and Health. 2nd ed. Switzerland: Springer; 2019. p. 207-25.
39. Austin PC, Tu JV. Bootstrap Methods for Developing Predictive Models. The American Statistician. 2004;58(2):131-7.
40. Tibshirani R. Regression Shrinkage and Selection Via the Lasso. Journal of the Royal Statistical Society: Series B (Methodological). 2018;58(1):267-88.
